# Favourable outcome after late reorientation of an upside-down Descemet Membrane Endothelial Keratoplasty (DMEK) graft: a case report

**DOI:** 10.1186/s12886-019-1181-3

**Published:** 2019-07-29

**Authors:** Siegfried Mariacher, Martina Mariacher, Karl Thomas Boden, Peter Szurman, Kai Januschowski

**Affiliations:** 1 Knappschaft Eye Clinic, Knappschaft Hospital Saar GmbH, An der Klinik 10, 66280 Sulzbach, Germany; 2 Centre for Ophthalmology, University Eye Hospital Tuebingen, Schleichstr. 12, 72076 Tuebingen, Germany

**Keywords:** DMEK, Upside-down, Inversion, Graft failure, Re-bubbling, Graft reorientation procedure

## Abstract

**Background:**

Clinical outcome after successful reorientation of an upside-down implanted DMEK (Descemet Membrane Endothelial Keratoplasty) graft 4 weeks after initial transplantation.

**Case presentation:**

A 71-year-old woman presented with Fuchs’ endothelial corneal dystrophy for DMEK. After initial DMEK the donor graft was fully attached and well centred during intracameral gas filling. When the gas bubble was fully resorbed the graft started to detach. Therefore, two intracameral gas injections were consecutively performed. During the second re-bubbling, an upside-down orientation was observed and so the graft was flipped, centred, re-attached and finally stabilized by an intracameral gas bubble. Three weeks after reorientation slit lamp examinations showed a well centred and attached graft, endothelial cells that started functioning and a patient’s visual acuity of 20/40. Visual acuity increased to a 20/32 vision in the observed eye three months later and further improved to 20/20 6 months after reorientation and stayed stable between 20/32 and 20/20 during the remaining 15 months of follow-up, with a clear and well-attached graft.

**Conclusion:**

Reorientation of an upside down DMEK graft was successful even 4 weeks after initial DMEK. Visual recovery and endothelial cell count increase were stepwise noticed during the first 6 months and 15 months after reorientation, respectively. Finally a favourable outcome with 20/32 to 20/20 vision at least 6 months after graft reorientation was achieved. Therefore, restoring full graft function could last several weeks or even months following (late) reorientation of an upside-down DMEK graft.

## Background

In Fuchs’ endothelial corneal dystrophy (FECD) or other corneal endothelial disorders reduction of postmitotic endothelial cells results in a loss of cellular hexagonal morphology (pleomorphism, polymegathism) and alteration in cell size. As a result of those alterations, the remaining endothelial cells are not able to maintain the normal pumping and leaking function. Corneal thickness and glare sensitivity increase, while visual clarity decreases.

Over the past years, Descemet Membrane Endothelial Keratoplasty (DMEK) has evolved rapidly as a preferable alternative to penetrating keratoplasty in patients with endothelial cell disorders like FECD. DMEK enables selective transplantation of the endothelial monolayer including the Descemet membrane, promising favourable functional and anatomical results [1].

Graft detachment is a potential complication after DMEK. Therefore, surgical interventions like air/gas re-injection inside the anterior chamber (graft re-bubbling) or graft reorientation of an upside-down graft can be considered. In cases of a detached graft and an assumed proper orientation of the transplant re-bubbling is the first treatment option to manage partially detached grafts after DMEK. The most important requirement for a successful reattachment is a correctly orientated graft [2].

A large retrospective multicentre real-life study investigated 2485 eyes after DMEK and found a re-bubbling rate of 20.1% [1]. During re-bubbling of a detached graft, the size of the bubble influences the outcome. Circovic et al. found that air bubbles of an 80% anterior chamber volume compared to a 50% anterior chamber volume decreased the risk of graft re-detachment after re-bubbling significantly from 27.0 to 8.1%, respectively [3].

Dirisamer et al. investigated the type of graft detachment in 150 consecutive DMEK cases and described clinical signs of an upside-down orientation of the graft like persistent stromal oedema and spike-shaped fibrous lesions between the donor and recipient interface [2].

This is the first report describing a successful graft reorientation of an upside-down DMEK graft even 4 weeks after initial DMEK.

## Case presentation

A 71-year old woman presented to our eye clinic with bilateral FECD. The patient’s medical history revealed previous cataract surgery. No other prior ocular surgery was performed in the past and no other ocular diseases were known. The woman had polymyalgia rheumatica and psoriasis arthritis treated with oral prednisolone 5 mg (Prednisolon ratio 5 mg, Ratiopharm GmbH, Germany) once a day combined with oral pantoprazole 40 mg (Pantozol 40 mg, Takeda GmbH, Germany) and subcutaneous adalimumab 40 mg (Humira 40 mg, Cc-Pharma GmbH, Germany) biweekly. Due to a previous stroke without any residual deficits, the patient was treated with aspirin 100 mg daily (ASS Hexal 100 mg, Hexal AG, Germany).

Best-corrected distance visual acuity (BCDVA) was assessed in the decimal scale and converted to Snellen equivalent for reasons of better comprehensibility and clarity. The preoperative BCDVA was 20/63 in her right eye and 20/63 in her left eye.

**Fig. 1:**
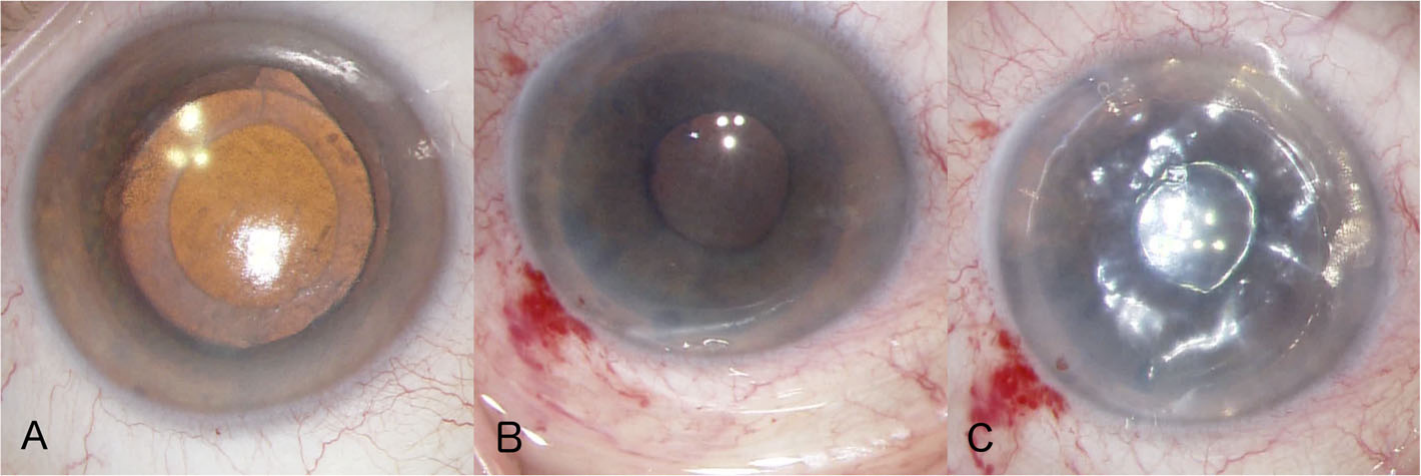
**a** Picture prior Descemet Membrane Endothelial Keratoplasty (DMEK). **b** Picture during implantation after unrolling the graft in the anterior chamber. **c** Picture directly after sufficient DMEK with an intracameral gas bubble

DMEK was performed on the right eye using a liquid bubble dissection technique [4] for lenticular preparation by injection of trypan blue into the sub-Descemet space via a sub-Descemet tunnel creating an enlarging liquid bubble. This bubble easily detached the Descemet membrane within a few seconds. Prior to implantation the donor cornea was trephined (8.25 mm) and loaded into a DMEK cartridge (Geuder GmbH, Germany) for intracameral injection. The graft exhibited 2380 endothelial cells per square millimetre, measured before stripping. After performing a peripheral iridectomy in the right recipient eye, the recipient Descemet membrane was stripped. The peripheral iridectomy was performed using intraocular microscissors The endothelium and Descemet’s membrane of the recipient’s cornea was stripped away through a corneal incision along an 8.25 mm demarcation line under balanced salt solution. The stripping was performed using an incision hook with irrigation. The diameter of the descemetorhexis was equal to the diameter of the Descemet membrane transplant. The donor tissue was then injected into the anterior chamber followed by double verification of the orientation of the graft. For double verification, the donor roll was placed in the injector, where it should be facing up and the edges of the DMEK graft should be facing downward. The double verification was made when the graft was located in the anterior chamber by using the Moutsouris sign [5]. After unfolding and positioning of the graft, an air-gas mixture bubble (Easygas SF6, Geuder GmbH, Germany) was injected into the anterior chamber. A mixture of 20% sulphur hexafluoride (SF6) and 80% air was used. During surgery, no complications or abnormalities were noted (Fig. 1).

**Fig. 2:**
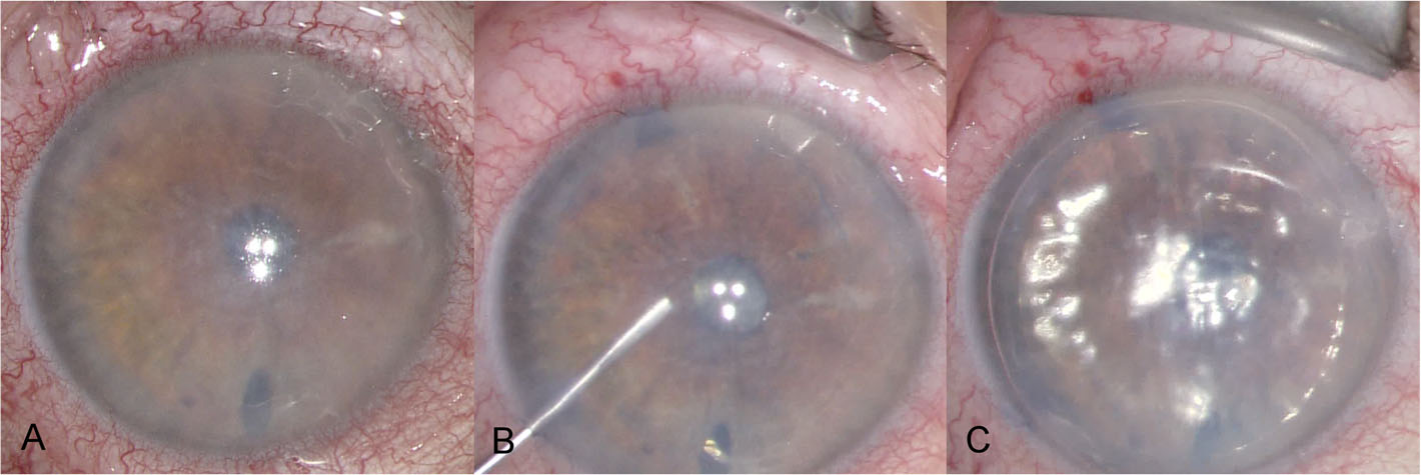
**a** Picture prior reorientation of the upside-down DMEK graft. **b** Picture after graft reorientation of the previous upside-down graft. **c** Picture at the end of reorientation with an intracameral gas bubble

The patient was instructed to maintain a supine position as long as the gas bubble was present. Examinations revealed a well-positioned transplant with a clear centre and only few Descemet’s membrane folds without any signs of detachment under the gas bubble on the first day after surgery. During the following days, most of the gas tamponade was absorbed, and progressive stromal oedema observed. Descemet’s membrane folds and subtotal Descemet detachment have occurred. A second air-gas mixture bubble was injected inside the anterior chamber on the sixth day after the initial surgery. After re-bubbling the patient showed a stable, well-centred graft with an air-gas bubble inside the anterior chamber and was discharged home. Three weeks after initial surgery the patient presented with blurred vision (BCDVA counting fingers), stromal corneal oedema, Descemet’s folds and Descemet’s membrane detachment infero-temporally. We discussed our findings with the patient and the shared decision was made to perform a second re-bubbling with controlling the orientation of the graft. Twenty-nine days after initial DMEK the patient presented for the planned intervention. Prior to the second re-bubbling, the patient achieved 20/600 vision in the right eye and presented with unchanged corneal findings. Before the injection of another air-gas mixture bubble, the surgeon noticed the unusual behaviour of the graft. The graft was detached from the cornea and checked again for an upside-down orientation. This was done by looking at the double roll of the graft which seemed to face downwards and so the surgeon concluded that the graft could be in an upside-down position. Therefore, the surgeon reorientated the graft by inversion of the entire transplant and injected the air-gas mixtureinside the anterior chamber after unfolding the graft (Fig. 2). Examinations on the first day after the surgical graft reorientation, what was 4 weeks after initial DMEK transplantation, showed a graft with a clearer centre compared prior to reorientation, only few Descemet’s membrane folds and the tendency to localized re-detach at the nasal edges.

**Fig. 3:**
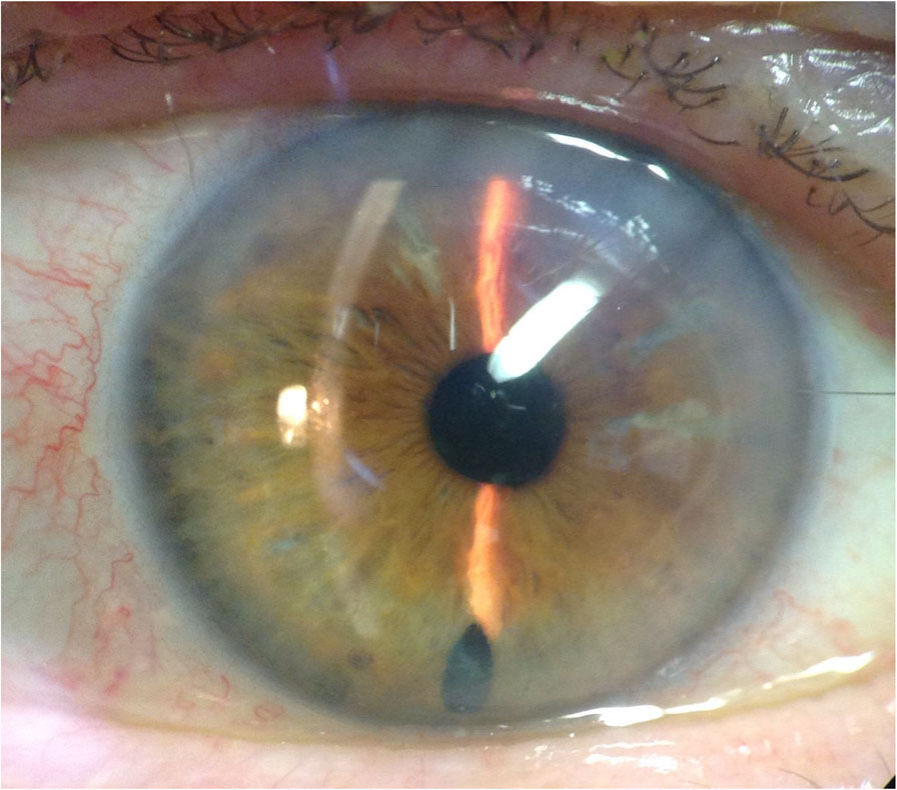
Slit lamp picture of the anterior segment 15 months after surgical graft reorientation of the previous upside-down transplant graft

**Fig. 4:**
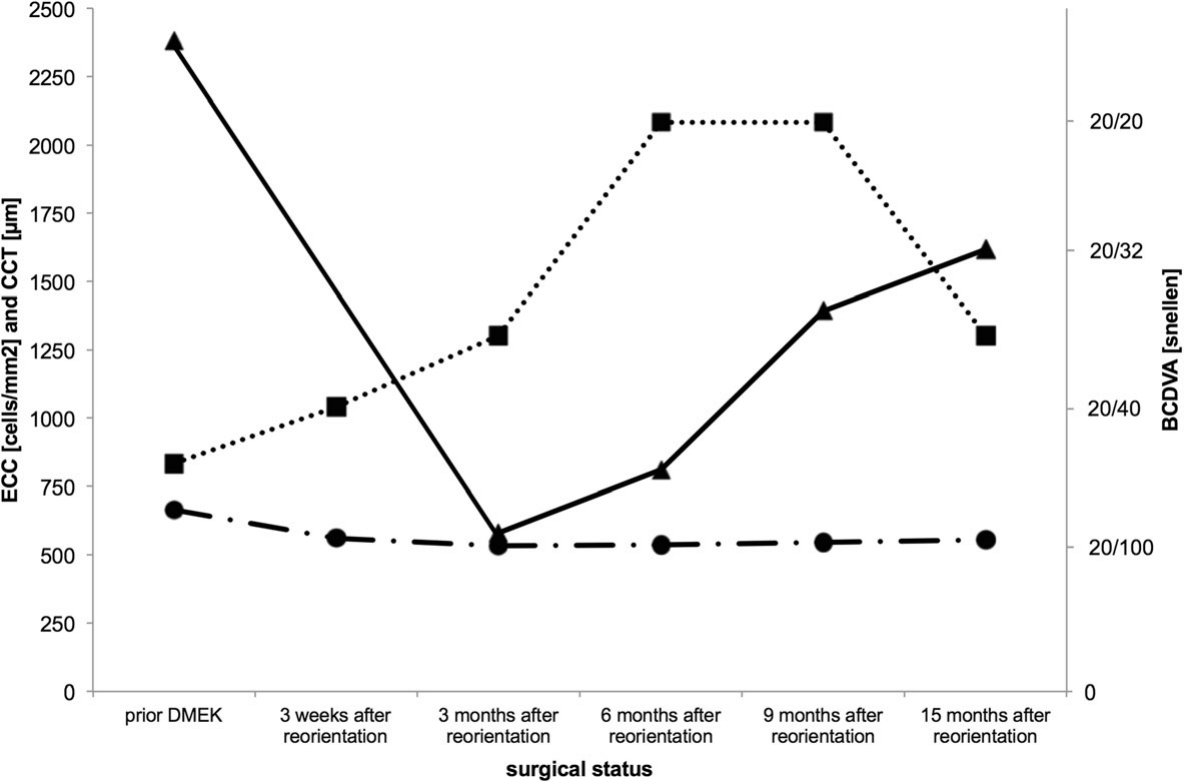
Best corrected distance visual acuity (BCDVA, dotted line, squares), endothelial cell count (ECC, solid line, triangles) and central corneal thickness (CCT, dash-dotted line, circles) prior initial DMEK and after reorientation of the upside-down DMEK graft

The patient then presented 3 weeks after reorientation of the upside-down DMEK with a 20/40 vision in the right eye, only slight Descemet’s membrane folds, clear corneal centre and without graft detachment. Further on the patient achieved 20/32, 20/20, 20/20, and 20/32 BCDVA 3, 6, 9, and 15 months after reorientation of the upside-down graft, respectively (Table 1; Figs. 3 and 4).

**Table 1:** Best Corrected distance visual acuity (BCDVA), endothelial cell count (ECC) and central corneal thickness (CCT) prior initial DMEK, prior and after first rebubbling and after second rebubbling and reorientation of the upside-down graft of the right eye

Surgical status	BCDVA	ECC [cells/mm^2^]	CCT [μm]
prior DMEK	20/50	graft: 2380	665
3 weeks after reorientation of the graft	20/40	N/A	560
3 months after reorientation of the graft	20/32	580	533
6 months after reorientation of the graft	20/20	811	536
9 months after reorientation of the graft	20/20	1392	545
15 months after reorientation of the graft	20/32	1618	555

## Discussion and conclusion

This is the first report concerning reorientation of an upside down DMEK graft several weeks after the initial transplantation. We were able to show a slow but distinctive functional and anatomical rehabilitation with surprisingly good visual outcome even 15 months after graft reorientation.

It is known that aqueous humour plays a major role in the immunosuppressive microenvironment of the anterior chamber. If the aqueous humour lacks specific immunosuppressive molecules, the immune privilege is abolished and corneal grafts become rejected [6]. Since the patient was under systemic immunosuppressive agents for her rheumatoid diseases, this could have played a substantially supportive role in the acceptance and survival rate of the corneal transplant. Even though systemic immunosuppressive therapy is well known in high-risk full-thickness corneal transplantation, there has been no clear data existing regarding immunosuppressants in DMEK grafts.

Grafts with lower endothelial cell counts are more likely to require additional air or gas re-injection. After uncomplicated DMEK endothelial cell count decreases between 19 and 33% 6 months after surgery [7, 8]. Mean endothelial cell loss is higher after re-bubbling compared to uncomplicated DMEK without the need for further air or gas instillation. Significantly more endothelial cell loss occurred in eyes with two or more re-bubbling interventions compared to only one re-bubbling procedure [9].

In our presented case central endothelial cell count measurements increased very slowly after the reorientation of the graft. Six months after surgery the number of endothelial cells per square millimetre was 66% lower than prior transplantation. Due to the slow but constant increasing cell count during the following months, reduction of the central endothelial cell count decreased to 32% 15 months after surgery. This is a remarkable finding. Comparison between 15 months data of our presented case and 12 months data of the above-mentioned studies [8] revealed only 10% higher (+ 4 to − 10%) endothelial cell count reduction in our case 15 months after reorientation compared to the measurements after uncomplicated DMEK. However, endothelial cell count improved very slowly after the 4 weeks of upside-down orientation of the graft. During the entire follow-up (15 months) the endothelial cell count showed significant improvement.

Visual acuity improved from 20/600 vision prior reorientation to 20/40 vision 3 weeks, 20/32 vision 3 months and 20/20 vision 6 months after surgery. So visual recovery lasted longer than previously expected and even 6 months after reorientation further improvement in visual acuity was observed. A prolonged period over 6 months was required for the graft to regain complete function.

Upside-down orientation was first noticed due to unusual behaviour of the graft during the second intracameral air-gas mixture injection. Our presented patient showed a marked reduction in corneal clearance with reduced visual acuity but no spike shaped fibrous lesions as described by Dirisamer et al. [2](Fig. 2a).

Marked reduction in corneal clearance, increased corneal thickness, and a reduced visual acuity should be considered as a sign of the upside-down orientation of the graft.

Fernandez Lopez described a higher rate of graft detachment, increased graft stiffness and/or fibrosis when re-bubbling was performed several weeks after initial DMEK [10]. In our patient, the reorientation of the upside-down graft 4 weeks after implantation was not affected by any complications. The graft was handled without any problems during reorientation and no visual damage of the graft could be found at the end of surgery.

An upside-down orientation of the DMEK should also be considered if re-bubbling is not sufficient and an inverse corneal clearance pattern is observed. Our case demonstrated, that even 4 weeks after initial DMEK a successful graft reorientation is a possible treatment option with good visual outcome (BCDVA of 20/20 vision; 6 months after surgery).

## Data Availability

All data are presented in the manuscript and Figures.

## Abbreviations

BCDVA: Best corrected distance visual acuity
DMEK: Descemet membrane endothelial keratoplasty
FECD: Fuchs’ endothelial corneal dystrophy

## References

[1] Oellerich S, Baydoun L, Peraza-Nieves J, Ilyas A, Frank L, Binder PS (2017). Multicenter study of 6-month clinical outcomes after Descemet membrane endothelial Keratoplasty. Cornea..

[2] Dirisamer M, van Dijk K, Dapena I, Ham L, Oganes O, Frank LE (2012). Prevention and management of graft detachment in descemet membrane endothelial keratoplasty. Arch Ophthalmol Chic Ill 1960.

[3] Ćirković A, Beck C, Weller JM, Kruse FE, Tourtas T (2016). Anterior chamber air bubble to achieve graft attachment after DMEK: is bigger always better?. Cornea..

[4] Szurman P, Januschowski K, Rickmann A, Damm L-J, Boden KT, Opitz N (2016). Novel liquid bubble dissection technique for DMEK lenticule preparation. Graefes Arch Clin Exp Ophthalmol Albrecht Von Graefes Arch Klin Exp Ophthalmol.

[5] Dapena I, Moutsouris K, Droutsas K, Ham L, van Dijk K, Melles GR (2011). Standardized "no-touch" technique for descemet membrane endothelial keratoplasty. Arch Ophthalmol.

[6] Streilein JW (2003). New thoughts on the immunology of corneal transplantation. Eye..

[7] Goldich Y, Showail M, Avni-Zauberman N, Perez M, Ulate R, Elbaz U (2015). Contralateral eye comparison of descemet membrane endothelial keratoplasty and descemet stripping automated endothelial keratoplasty. Am J Ophthalmol.

[8] Ham L, van Luijk C, Dapena I, Wong TH, Birbal R, van der Wees J (2009). Endothelial cell density after descemet membrane endothelial keratoplasty: 1- to 2-year follow-up. Am J Ophthalmol.

[9] Feng MT, Price MO, Miller JM, Price FW (2014). Air reinjection and endothelial cell density in Descemet membrane endothelial keratoplasty: five-year follow-up. J Cataract Refract Surg.

[10] Fernández López E, Baydoun L, Gerber-Hollbach N, Dapena I, Liarakos VS, Ham L (2016). Rebubbling techniques for graft detachment after Descemet membrane endothelial Keratoplasty. Cornea..

